# mTOR Controls Ovarian Follicle Growth by Regulating Granulosa Cell Proliferation

**DOI:** 10.1371/journal.pone.0021415

**Published:** 2011-07-05

**Authors:** James Yu, Aylin Yaba, Corinna Kasiman, Travis Thomson, Joshua Johnson

**Affiliations:** 1 Department of Obstetrics, Gynecology, and Reproductive Sciences, Yale School of Medicine, New Haven, Connecticut, United States of America; 2 Department of Histology and Embryology, Akdeniz University, Antalya, Turkey; 3 Smith College, Northampton, Massachusetts, United States of America; 4 Program in Molecular Medicine and Program in Cell and Developmental Dynamics, University of Massachusetts Medical School, Worcester, Massachusetts, United States of America; Stanford University, United States of America

## Abstract

We have shown that inhibition of mTOR in granulosa cells and ovarian follicles results in compromised granulosa proliferation and reduced follicle growth. Further analysis here using spontaneously immortalized rat granulosa cells has revealed that mTOR pathway activity is enhanced during M-phase of the cell cycle. mTOR specific phosphorylation of p70S6 kinase and 4E-BP, and expression of Raptor are all enhanced during M-phase. The predominant effect of mTOR inhibition by the specific inhibitor Rapamycin (RAP) was a dose-responsive arrest in the G1 cell cycle stage. The fraction of granulosa cells that continued to divide in the presence of RAP exhibited a dose-dependent increase in aberrant mitotic figures known as anaphase bridges. Strikingly, estradiol consistently decreased the incidence of aberrant mitotic figures. In mice treated with RAP, the mitotic index was reduced compared to controls, and a similar increase in aberrant mitotic events was noted. RAP injected during a superovulation regime resulted in a dose-dependent reduction in the numbers of eggs ovulated. Implications for the real-time regulation of follicle growth and dominance, including the consequences of increased numbers of aneuploid granulosa cells, are discussed.

## Introduction

Large scale clinical investigations have begun to reveal that dietary and/or lifestyle choices can correlate with ovulatory fertility [1]–[4]. Except for the striking example of functional hypothalamic amenorrhea reported by Berga and colleagues [5], [6], mechanisms by which ‘environmental’ stressors and nutritional status affect reproductive function have been elusive. Indeed, the majority of anovular/amenorrhoeic patients suffer for occult reasons. We hypothesize that the mammalian Target Of Rapamycin) Serine/Threonine kinase (mTOR) is a conserved, critical factor in the production of ‘healthy’ eggs capable of giving rise to offspring.

mTOR acts to integrate signals from mitogens, stress, and available nutrition [7], [8]. During periods of compromised nutrition (decreased available amino acids or sugars), growth factor withdrawal, or stress, mTOR activity is decreased. This leads to reduced cell proliferation [9] and tissue growth [10], and the onset of autophagy [11]. mTOR functions as part of at least two multi-protein complexes, each of which has defined roles in the control of cell growth.

The first TOR complex, the mammalian TOR complex 1 (mTORC1) consists of mTOR and cofactor proteins including Raptor [12]–[14]. The other complex, mTORC2, includes the cofactor Rictor in place of Raptor [15], [16]. mTORC2 has been shown to regulate the organization of the actin cytoskeleton, acting through Rho GTPases [15], [17]. The namesake inhibitor Rapamycin (RAP) mimics the effects of cell stress or nutrient starvation by blocking mTOR directly after binding an intracellular receptor, FKBP12. The FKBP12-RAP complex was originally thought to only inhibit mTORC1 signaling [18], however, inhibitory effects on mTORC2 have been uncovered [19]–[21], suggesting that metabolic and cytoskeletal regulation by mTOR are tightly integrated.

mTOR controls the initiation of protein translation. Under non-stressed conditions, mTOR phosphorylates p70 S6 kinase (p70S6K) and eukaryotic translation initiation factor 4E-binding protein 1 (4E-BP1). A lack of mTOR phosphorylation of p70S6K results in reduced downstream action upon the 40S ribosomal protein S6 and eIF4B, each of which recruit ribosomes to the 5′ end of an mRNA [22]. In 2004, Alam et al. [7] showed that p70S6K phosphorylation in primary rat granulosa cells is dependent on mTOR activity, confirming that this part of the pathway is intact in granulosa cells. 4E-BP1 is a translational repressor that acts by binding the eIF4E translation initiation factor. Phosphorylation of 4E-BP1 by mTOR disrupts this interaction, freeing eIF4E to initiate cap-dependent translation [23], [24].

Endocrine, paracrine, and autocrine signaling all converge on granulosa cells, controlling their growth and differentiation [7], [25], [26]. We have shown that mTOR inhibition in primary mouse granulosa cells and follicles results in reduced granulosa cell proliferation *in vitro* [8]. Using the *Drosophila* model system, we have also detected specific effects upon ovarian function *in vivo*. In flies, ingestion of RAP results in dose-dependent interruption of oogenesis, effectively sterilizing females [27] (see [28] for a review). However, it remained to be seen whether inhibition of mTOR *in vivo* in mammals would affect ovarian function.

Here, we took advantage of the the convenient spontaneously immortalized rat granulosa cell line (SIGC) [29]. SIGC were originally derived by culturing BD IV rat granulosa cells from punctured follicles *in vitro*, and isolating those cells that continued to proliferate after approximately 13 passages (instead of luteinizing). Banu et al. [30] performed a detailed gene expression analysis of the cells, showing that the cells maintained a granulosa identity (expressing Estrogen Receptor-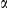 and -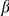, Follicle Stimulating Hormone Receptor, Luteinizing Hormone Receptor, Steroidogenic Factor-1, and other functional markers of granulosa cells). Interestingly, the group that originally established SIGC lines found that while in early passages, the cells were almost uniformly diploid, after extended passages, the cells became tetraploid [29].

mTOR's action during proliferation was originally thought to be limited to the mitogen-sensitive G1 stage [31], [32] of the cell cycle. However, evidence is building for M-phase specific roles for mTOR signaling [8], [33]. Indeed, an autophosphorylated form of mTOR has been localized to midzone microtubules and the midbody of the mitotic apparatus in cancer cells [34]. In the studies presented here, preliminary examination showed that expression of mTOR signaling pathway molecules in SIGC was similar to that seen in mouse granulosa cells *in vivo*, including during M-phase. We thus used the cell line to evaluate the effects of mTOR inhibition upon proliferation, cell cycle stages, and mitotic events. To determine the effects of mTOR inhibition *in vivo*, mouse follicle development and ovulation were measured in the presence and absence of RAP.

## Results

### mTOR signaling molecule expression is conserved in spontaneously immortalized rat granulosa cells

**Figure 1:**
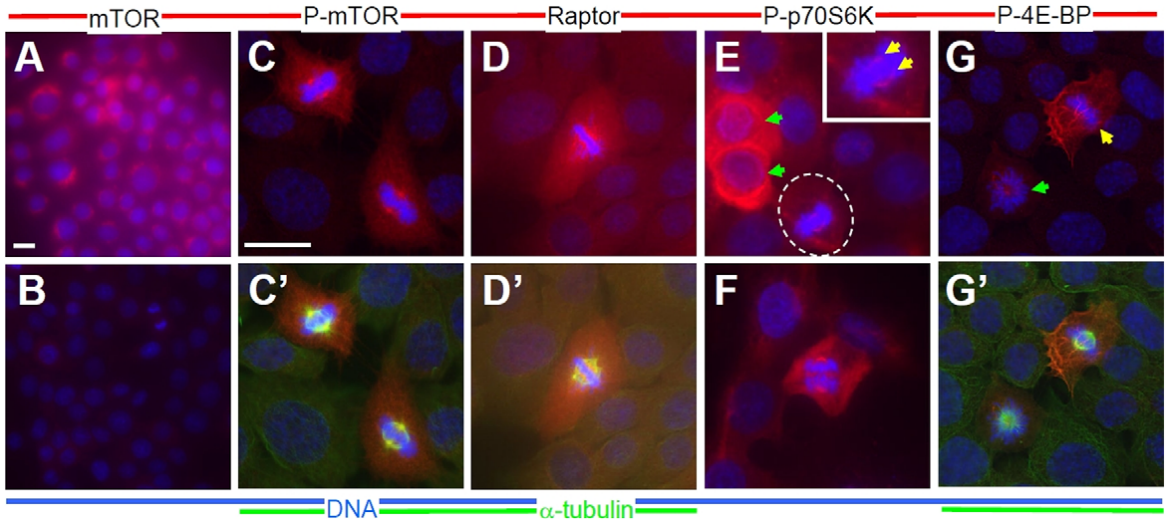
Expression of mTOR signaling molecules in Rat SIGC. Compared to total mTOR (A), phospho-Ser2448 mTOR (C,C′) is upregulated during M-phase (see also Fig. S1). Cells processed identically except for omitted first antibody are shown in (B). Raptor (D) is expressed at higher levels during mitosis relative to neighboring interphase cells. Phosphorylation of the downstream mTOR substrates phospho-Thr 389 p70S6K (E,F), and phospho-Thr 37/46 4E-BP (G) is also enriched during mitosis. P-p70S6K in particular is localized to regions adjacent to chromosomes (E, dashed oval and inset, yellow arrows). Phospho-p70S6K was also enriched in approximately 1% of interphase cells (E, green arrows). Co-staining with 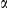-tubulin reveals that P-mTOR (C′), Raptor (D′), and P-4E-BP (G′) are present at high levels in the region of the mitotic spindle.

First, we asked whether the expression of mTOR signaling molecules was conserved between mouse ovary, primary mouse granulosa cells [8], and SIGC (Fig. 1). We extended our studies to include the cofactor Raptor and two downstream mTOR kinase targets, p70S6 kinase and 4E-BP. Care was taken to evaluate mTOR signaling molecule expression during interphase and during the individual stages of M-phase. As in mouse granulosa cells, mTOR is expressed at consistent levels in the cytoplasm in SIGC (red, Fig. 1A; compare to no-first-antibody control shown in 1B). Again as seen in mouse granulosa cells, Serine 2448-phosphorylated mTOR (P-mTOR) is very highly expressed during the mitotic G2/M cell cycle stage(s) (expression during metaphase of mitosis shown in Fig. 1C and 1C′; see Fig. S1 for representative expression from late G2/prometaphase through telophase). This phosphorylation of mTOR at Serine 2448 has been shown to correlate with mTOR kinase activity [35]–[37]. Co-staining for 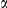-tubulin and P-mTOR revealed that the phosphoprotein was highly enriched in the region of the mitotic spindle (1C′).

Raptor, the cofactor required for RAP-sensitive mTORC1 kinase activity, was also expressed at high levels in SIGC during mitosis, also in the region of the mitotic spindle (Fig. 1D, 1D′ shows co-localization with the mitotic apparatus). After detecting both P-mTOR and Raptor in this region, we sought to determine whether known downstream mTOR kinase substrates might be upregulated during mitosis, potentially in the region of the mitotic spindle. Both phospho Threonine 389 p70 s6 kinase (P-p70S6K, red channel, Figs. 1E, F) and phospho Threonine 37/46 4E-BP (P-4E-BP, red, Figs. 1G, G′) were enhanced in mitotic cells. As with P-mTOR and Raptor, P-p70S6K was enriched in the region of the mitotic spindle adjacent to metaphase chromosomes in these granulosa cells (1E, cell in oval shown in magnified inset, 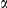-tubulin staining omitted to highlight narrow bands of P-p70S6K expression-red, denoted by yellow arrows, adjacent to chromosomes-blue). P-p70S6K was also expressed at high levels in a subset of SIGC in interphase (1E, green arrows). In 1F, an early anaphase cell is shown, where P-p70S6K remains expressed at high levels in the region of the mitotic spindle. The expression of P-4E-BP was enhanced during mitosis (arrows, green arrow-cell in prometaphase, yellow, metaphase, G′ shows staining of P-4E-BP overlaid with 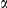-tubulin), and expression in the region of the mitotic spindle and throughout cells was found (G′). Overall, the expression of mTOR was found to be similar to that seen in granulosa cells of the mouse ovary, and the expression of signaling pathway members and substrates suggested a targeting of this pathway to the mitotic spindle in SIGC.

Comparison of ‘active’ P-mTOR and cells that express high levels of Raptor and the substrates suggest a high degree of overlap within the cell cycle, specifically within M-phase. While P-mTOR is present at high levels between late G2 (early stages of chromosome condensation) and mitotic telophase, Raptor has a tighter window of expression (1D and Fig. S1). High Raptor expression begins during late G2 and ends at approximately mitotic anaphase. This can be seen by comparing the entirely consistent expression of P-mTOR during anaphase (Fig. S1A, yellow arrows) and beyond to cytokinesis (Fig. S1A, yellow arrowhead) with Raptor, the expression of which is inconsistent during anaphase (Fig. S1B, compare anaphase mitotic figure at yellow arrow with those marked by white arrowheads) and is not seen during late anaphase/telopase/cytokinesis. This rapid disappearance of Raptor is suggestive of regulated degradation during and after mitosis and warrants further study. The mTOR substrates P-Ser p70S6K and P-Thr 37/46 also showed enriched expression between late G2 and anaphase. The only exception to the overlap of expression of mTOR, Raptor, and the substrates, was an interesting, strong predominantly cytoplasmic expression seen in approximately 1% of interphase SIGC (Fig. 1G, green arrowhead). The conserved expression patterns relative to the mouse [8] supported the use of the SIGC cells as a model for studies of mTOR function during granulosa survival, mitosis, and proliferation.

### mTOR inhibition results in altered granulosa cell proliferation

**Figure 2:**
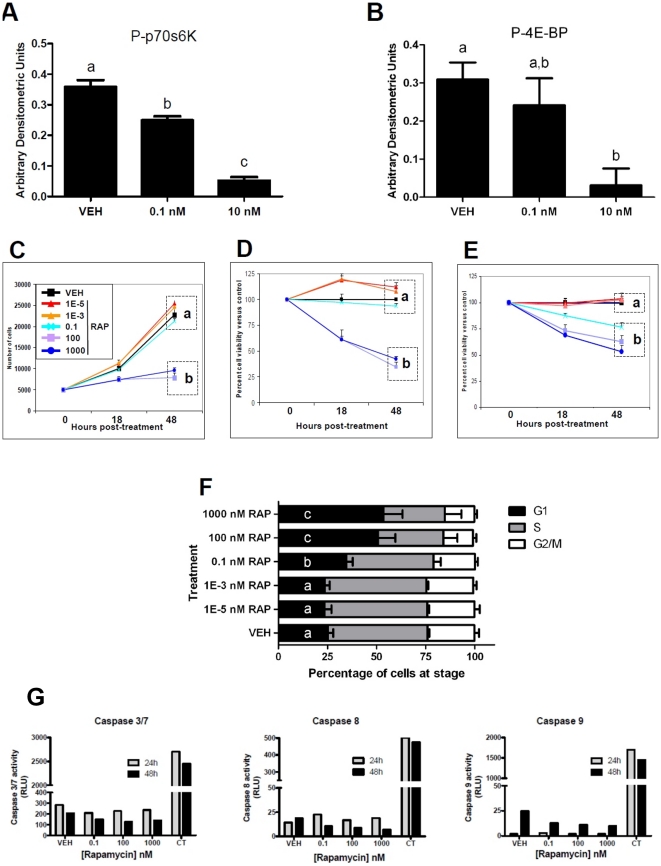
mTOR inhibition by RAP in Rat SIGC. Treatment with RAP is shown to inhibit mTOR activity by reducing phosphorylation of the target residue of p70S6 kinase (A) and 4E-BP (B). As low as 0.1 nM RAP significantly (P
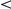
0.05, one-way ANOVA, letters denote bars that significantly differ from one another) reduces P-p70S6K and P-4E-BP eighteen hours after treatment. Cells were similarly treated with a dose curve of RAP (C–E) and were counted (C) or processed for biochemical viability assay (D) at 18 and 48 hours post-treatment. High doses (100 and 1000 nM) of the drug resulted in significantly (P
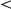
0.0001, one way ANOVA with Bonferroni's Multiple Comparison Test, denoted a, b) reduced cell number and viability at 48 hours compared to all other treatments (denoted by a,b). Low doses (1E-3 and 1E-5 nM) of RAP resulted in non-significant but consistently increased cell number at 18 and 48 hours. Washout of RAP after 3 hours of treatment resulted in reduced, but still significant reductions in cell viability 48 hours post-treatment (E). Altered SIGC proliferation correlated with altered cell cycle parameters (F). Cells were grown in the dose curve of RAP. 18 hours post-treatment, cells were fixed and processed for DNA content cell cycle analysis via flow cytometry. Average results from three individual experiments are shown in F. As seen in other cell types, a dose-responsive induction of cells in the G1 stage of the cell cycle occurred with RAP treatment. Significantly different percentages of cells in G1 were found (P
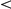
0.05, one way ANOVA with Bonferroni's Multiple Comparison Test, significantly different populations denoted by a,b,c) with increasing RAP. (G) RAP does not induce Caspase activity in Rat SIGC. SIGC were treated with either vehicle (VEH) or specified concentrations of RAP or Camptothecin (CT - positive control for apoptosis induction). Cells were collected at 24 and 48 hours post-treatment and processed for plate based chemiluminescent Caspase activity measurements. Relative Luminescence units are graphed for each sample per time point. Data shown are the average of two unique experiments.

Prior to testing the effects of mTOR inhibition upon SIGC proliferation, we confirmed that RAP indeed inhibited kinase activity in these cells. We treated SIGC with a concentration series of RAP and assessed the expression of P-p70S6 kinase (Thr 389) and P-4E-BP (Thr 37/46) by Western blot (Fig. 2A,B). SIGC cells were either treated with ethanol vehicle, 0.1 nM RAP, or 10 nM RAP for 18 hours. mTOR inhibition was confirmed as P-p70S6K (2A) and P-4E-BP (2B) were each significantly reduced, in respective dose-dependent fashions (graphed as a ratio of target band density to beta actin loading control band density) in the presence of RAP.

We then assessed cell number (Fig. 2C) directly and indirectly (*via* biochemical assay, Fig. 2D) 18 and 48 hours post-treatment with either ethanol vehicle or a concentration series of RAP. As cell death was not induced at any concentration of RAP used, the cell ‘viability’ assays measured relative cell proliferation rates and were not reflective of cells lost to toxicity. A biphasic response was seen across the concentration series. That is, higher concentrations of RAP (100 and 1000 nM) resulted in significantly reduced proliferation at 18 and 48 hours versus controls, and, low concentrations (here, 1E-3 and 1E-5 nM) of RAP resulted in increased proliferation versus vehicle. The mitogenic effect of low RAP was reminiscent of the effects seen on cultured mouse follicles in our previous study [8].

Next, we tested whether long term exposure to RAP was required for the effects upon SIGC proliferation. We treated SIGC with identical concentrations of the drug, washing it out after 3 hours, and again measured cell viability at 18 and 48 hours post-treatment onset. The effects of transient exposure are summarized in Figure 2E. Here, the overall effects of mTOR inhibition were lessened from that of constant treatment, but significant declines in viability were again found after treatment with the two highest doses of RAP. Interestingly, the increased cell proliferation and viability seen in cells with constant low levels of RAP (Fig. 2C,D) were not seen when the drug was washed out. This suggests that the mitogenic effect of low RAP requires longer, if not constant, exposure. Overall, mTOR inhibition resulted in striking effects upon granulosa cell proliferation and population size.

### mTOR inhibition alters granulosa cell cycle parameters

When inhibited by physiological cell stressors or by pharmacological means, mTOR inhibition has been shown by other groups to result in decreased proliferation mainly by inducing cell cycle arrest in the G1 stage. It should be noted that inhibition of mTOR during *interphase* would be responsible for arrest in G1, and thus the interphase expression patterns of P-mTOR, Raptor, P-p70S6K, and P-4EBP (Fig. 1) should not be ignored. Consistent with this, cell cycle analysis of SIGC by flow cytometry showed that significantly greater percentages of cells were present in the G1 fraction (Fig. 2F) in the presence of high RAP (approximately 55%), while lower percentages of cells were in G1 when low RAP or vehicle was present (approximately 25%). Not all cells were arrested in G1 in any treatment, that is, a fraction of cells always continued to divide. We confirmed the presence of actively mitotic cells by performing live-cell video measurements, which revealed that no significant difference between the length of time taken in mitosis (from ‘rounding up/prometaphase’ to complete cytokinesis) resulted from any treatment (Kasiman, Yu, and Johnson, unpublished). Interestingly, video microscopic observation of karyokinesis and cytokinesis revealed a fraction of cells whose chromosomes appeared “trapped” within the region of cell division, a feature that we later assessed in detail (below).

### Cell death is not induced by mTOR inhibition

In order to characterize the growth dynamics of SIGC cultures, we completed a number of concurrent studies to test whether cell death was being induced by RAP treatment. Gross hallmarks of cell death (e.g., lack of overt DNA condensation and blebbing) were absent from any cells processed for direct cell counts, viability measurements, and flow cytometry (2F), even when the super-physiological dose, 1000 nM RAP, was applied. We stained SIGC with Annexin V/Propidium iodide (data not shown) and performed biochemical caspase assays (Fig. 2G) to test for the induction cell death. In sum, these experiments confirmed that neither apoptosis nor necrosis resulted from any dose of RAP used. Due to the detection of mTOR signaling pathway molecules in the region of the mitotic spindle (Fig. 1) and the video microscopic observation that mitotic anomalies seemed to follow RAP treatment, we went on to carefully characterize mitotic figures in treated versus control cells.

### Anaphase bridges are consistently induced by mTOR inhibition; estradiol consistently decreases the percentage of anaphase bridges

**Figure 3:**
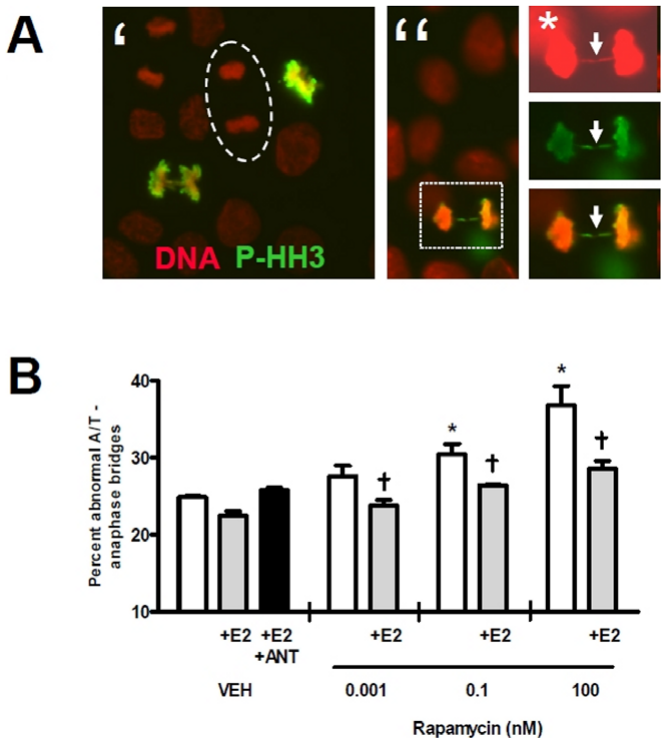
Mitotic errors and chromosomal aneuploidies are induced by RAP in a dose-dependent fashion; Estradiol consistently reduces mitotic anomalies. SIGC for were grown for 18 hours in the presence or absence of RAP, and then were fixed and stained with DAPI (red) and optionally the mitosis-specific Phospho Histone H3 Ser10 chromatin modification (PHH3, green) using a fluorophore-conjugated antibody. Representative staining is shown in A′, and a ‘normal’ anaphase/telophase mitotic figure where chromosome segregation has occurred entirely is highlighted (encircled by oval). An abnormal mitotic figure demonstrating an anaphase bridge is shown in A″ (* denotes higher magnification inset where DNA signal has been amplified electronically at top right to completely reveal DNA bridge, PHH3 and merged images below). Abnormal anaphase/telophase mitotic figures were counted and the percent found per treatment are summarized in B. RAP was found to increase abnormal mitotic figures in a dose-dependent fashion, and significant increases were found at 0.1 and 100 nM drug (*, P
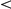
0.001 vs. vehicle-only (VEH) controls). Concurrent treatment with 10 ng/ml estradiol significantly reduced abnormal mitotic figures, 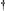 P
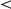
0.05, versus their individual corresponding RAP-only treatments.

As shown, despite the overall growth curves, consistent fractions of cells continue to divide in the presence of even the highest concentrations of RAP used. The only detectable difference between treated and untreated cells was an apparent increase in mitotic abnormalities in treated cells. This caused us to ask whether such abnormalities were being induced by mTOR inhibition. We hypothesized that the population of cells that continues to grow in the presence of RAP would experience abnormalities in mTOR signaling during mitosis and potentially, mitotic abnormalities, in a significant, dose-dependent manner. We went on to directly assess those continuing mitotic events (Fig. 3).

Assessment of mitotic figures in treated versus control cells was performed by staining cells with DAPI and mitosis-specific Phospho-Histone H3 (Ser10, denoted PHH3) (example of normal mitotic figures shown in Figure 3A′). While DAPI staining alone was sufficient for revealing the majority of bridges, PHH3 staining generally increased contrast (see electronically amplified DNA signal in Fig. 3A″, denoted by *, versus overlay) and made their characterization faster. This analysis revealed a surprisingly high representation of mitotic anomalies referred to as anaphase bridges [38], even in untreated cells. Anaphase bridges result where one or more pairs of sister chromatids fail to disjoin during mitosis and a strand of chromosome is trapped in the region of cytokinesis. The ultimate fate of most such bridges, if not resolved, is the degradation of the DNA within and thus the development of chromosomal aneuploidy. After 18 hours of treatment, we counted a minimum of four fields of 100 mitotic figures per treatment. This revealed that the defined mitotic abnormalities known as anaphase bridges and lagging chromosomes were indeed induced by treatment with RAP (percent abnormal anaphase/telophase anaphase bridges shown in Fig. 3B), in a dose-responsive manner.

Treatment with 0.1 and 100 nM RAP resulted in significantly (*, P
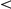
0.001) higher percent abnormal A/T mitotic figures than vehicle treatment. When considering the rates of aneuploid granulosa cells found in growing follicles, it is striking that dominant follicles have been shown to have fewer aneuploid granulosa cells [39]–[41]. As high estradiol production is also a feature of the dominant follicle, we hypothesized that estradiol would counter the induction of aberrant mitotic figures by RAP. When estradiol was included in cultures at 10 ng/ml (to approximate intrafollicular estradiol), the percent of abnormal anaphase/telophase figures versus corresponding RAP-only treatments was significantly reduced (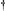, P
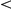
0.05). Interestingly, the inclusion of this concentration of estradiol slightly but insignificantly reduced the percent abnormal mitotic figures seen in vehicle treated cells in a manner that was countered by the addition of 100 nM ICI182780 estradiol receptor antagonist. Overall, these data prompted us to test whether similar effects of mTOR inhibition upon granulosa cell mitosis result within growing follicles *in vivo*.

### Mitotic abnormalities arise *in vivo* in response to mTOR inhibition

**Figure 4:**
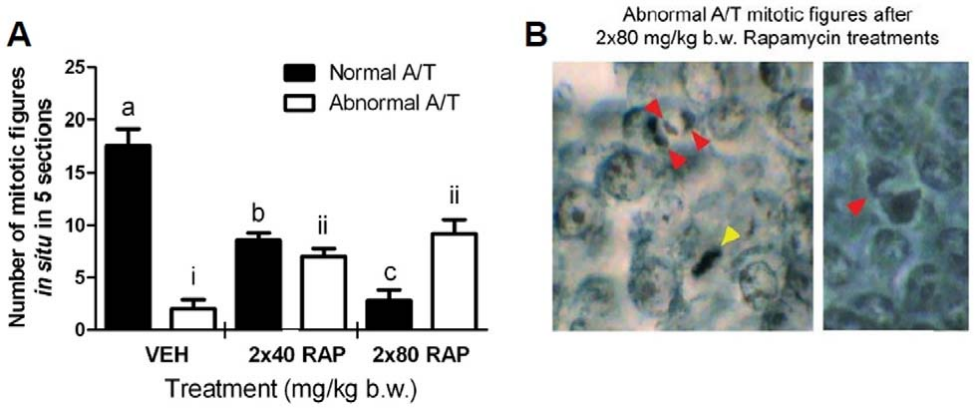
Mitotic errors are induced in granulosa cells of large preantral follicles by systemic RAP treatment in mice. Histological preparations of adult C57Bl/6 ovaries (n = 3 mice per treatment) collected 24 hours after the second of two RAP injections were analyzed for the total number of normal versus abnormal mitotic figures (A) in 5 follicles that contained between 10 and 15 granulosa cell layers. Significant differences were seen in both normal A/T mitotic figures (black bars denoted a,b,c; P
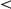
0.005, one way ANOVA with Bonferroni's Multiple Comparison Test) and abnormal figures (i,ii,iii). Examples of abnormal mitotic figures are shown in (B), where a normal A/T metaphase mitotic figure can be seen (yellow arrowhead) near a mitotic figure displaying a lagging chromosome (three red arrowheads denote anaphase chromosomes and lagging central chromatin). A representative anaphase bridge is shown at right.

In order to test whether mitotic abnormalities arise *in vivo* in follicles, we injected 8 week-old female mice on two consecutive days with vehicle or the doses of RAP specified in Fig. 4. Histological preparations were made from the ovaries and granulosa mitotic figures were counted within individual follicles from ‘end to end. ’ Fig. 4A shows results from counts of granulosa cell mitotic figures are shown along with examples of mitotic figures (Fig. 4B). As seen in SIGC treated *in vitro*, granulosa cells in follicles demonstrate a dose-responsive increase in abnormal anaphase-telophase mitotic figures in treated animals versus vehicle. Detecting this same effect *in vivo* supported the use of SIGC to approximate these processes and also suggests that the mitotic errors detected are likely to arise through similar processes as seen in the cell line. Even so, the use of RAP raised the possibility that broader non-physiological or toxic effects could result, inducing follicle atresia *in vivo*, perhaps even affecting the ‘primordial reserve’ of oocytes. We went on to assess this directly.

### Follicle reserve is unaffected by in vivo mTOR inhibition; RAP reduces ovulation in a dose-responsive manner

**Figure 5:**
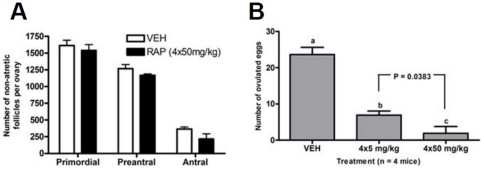
mTOR inhibition does not impact the primordial reserve of oocytes but does inhibit ovulation. Adult C57Bl/6 females were dosed with either vehicle or 4 daily does with 50 mg/kg b.w. RAP. Healthy follicles were counted in histological preparations of ovaries, results are summarized in A. No significant difference was found in either primordial follicles, or, growing preantral or antral follicles. Separately, adult animals recieved either 4 doses of vehicle or RAP beginning on day 1 of a superovulation regime. On day 4, ovulated eggs were collected, and their numbers are summarized in B.

To determine whether mTOR inhibition using systemic RAP delivery affected follicle health in mice, we used a direct histological approach [42], [43]. We found that numbers of healthy follicles were not affected by RAP delivery versus vehicle (Fig. 5A). RAP treatment thus does not result in detectable follicle loss *via* atresia. However, the numbers of ovulated eggs were reduced in a dose-responsive manner when RAP was included in a ‘superovulation’ regime (Fig. 5B, see Fig. S2 for injection regime). Significant differences (P
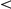
0.05) were seen between groups treated with 4 doses of vehicle, 5 mg/kg, or 50 mg/kg body weight. No difference in the number of eggs with fragmented or ‘unhealthy’ appearance was seen in RAP treated animals in these experiments: ovulated eggs were predominantly of ‘healthy’ appearance. Finally, we tested whether oocyte quality is compromised when animals were treated with four 5 mg/kg doses of RAP versus those treated with vehicle (treatment scheme summarized in Figure S2). Here, we fertilized eggs from each group *in vitro* and monitored their development to blastocyst. Table 1. summarizes the results from these experiments. Three animals either received the same 5 mg/kg regimen of RAP or vehicle during superovulation and eggs were again collected. Data is presented individually for animals treated with RAP, with the average number of healthy-appearing eggs and the number and percent that developed to blastocyst after *in vitro* fertilization. As shown, while animals treated with this dose of the drug ovulated fewer eggs than controls, the fraction of eggs with a healthy appearance and the percent that developed to blastocyst did not significantly differ from that of vehicle-treated animals. Thus mTOR inhibition with this dose resulted in reduced ovulation but little if any deleterious effect upon those oocytes that did ovulate.

**Table 1:** mTOR inhibition suppresses ovulation but does not affect subsequent embryo development.

Animal	Healthy Oocytes Ovulated	Number of Healthy Eggs Per Mouse
	(Unhealthy)	that Developed to Blastocyst (%)
**1**	6 (3)	5 (83%)
**2**	11 (1)	7 (64%)
**3**	7 (5)	5 (71%)
**Average RAP Tx**	8 (3)	5.7 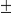 0.7 (72.7 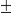 5.5%)
**Average VEH Tx**	20.3 (4)	16.1 (80 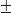 5.2%)

Groups of three 8-week old mice were injected with 4 doses of RAP or ethanol vehicle (VEH) during a superovulation regime. Eggs were collected and their appearance was evaluated (‘healthy’ or ‘unhealthy’). Healthy eggs were subjected to *in vitro* fertilization and development to blastocyst was scored.

## Discussion

In summary, mTOR inhibition resulted in reduced granulosa cell proliferation in the absence of an induction of cell death. This reduced proliferation results from an increased number of cells in the G1 stage of the cell cycle. Our data are consistent with reports showing that mTOR is involved in cell cycle progression at G1 [31], [32]. Some of the effects of mTOR inhibition on SIGC proliferation here can be seen occurring within the G2/M stage(s) of the cell cycle (Figs. 2, 3, 4). This outcome correlates with the detection of mTOR pathway molecules in the region of the mitotic apparatus [8], [33]. We have additionally detected increased fractions of cells that undergo anomalous mitotic events during mTOR inhibition.

As seen previously *in vivo* in the mouse ovary and *in vitro* in cultured primary mouse granulosa cells [8], the mTOR pathway appears to be upregulated during M-phase of the cell cycle. Here, SIGC cells were found to exhibit the same upregulation of Ser 248-phosphorylated mTOR, and also enhanced phosphorylation of the downstream targets p70S6 kinase and 4E-BP. Both mTOR targets demonstrated markedly enhanced expression near and around mitotic chromosomes. Interestingly, the mTORC1 cofactor Raptor appears to also be upregulated during M-phase. As we performed staining for the total protein (and not a phosphoprotein), it may be that the expression or stability of Raptor protein is altered before and during M-phase. Raptor also was found to be enhanced in the region of the mitotic spindle in SIGC cells. The recent finding that Raptor is phosphorylated by cdc2 prior to and during M-phase may be related to this [33] enhancement. Overall, enhanced mTOR complex and phosphoprotein targets, were found to be consistently higher in M-phase than during interphase.

The effects of RAP treatment of mice *in vivo* are consistent with effects on follicle growth to ovulatory stages. RAP treatment did not result in detectable effects on the pool of primordial follicles, nor upon the number of growing preantral follicles. However, the number of antral follicles was slightly reduced compared to vehicle-treated animals, and this corresponded to a reduction in the number of ovulated eggs. While compromised follicle development to ovulation might have also resulted in deleterious effects upon the eggs that were ovulated in the presence of RAP, the fraction of eggs that developed to blastocyst after fertilization was unchanged compared to vehicle.

The *in vivo* delivery of RAP may be acting at multiple levels in the mouse to limit the growth of maturing follicles. It is possible that mTOR inhibition in the hypothalamus or pituitary could lessen the production of gonadotrophin hormones, contributing the the effects within the ovary. However, our previous data showing that treatment of mouse follicles *in vitro* with RAP results in growth retardation [8] supports direct effects at the level of ovarian follicles. Because of this, mTOR's action *in vivo* in the ovarian follicle is supported as an important positive regulator of follicle growth and development. mTOR inhibition appears to slow the growth of the follicle in two ways: increased cell cycle arrest in the G1 stage of the cell cycle (as previously seen in several different cell types), and *via* the induction of mitotic anomalies in granulosa cells.

Those granulosa cells that continued to divide in the presence of high RAP are increasingly susceptible to mitotic abnormalities including anaphase bridges. Anaphase bridges arise where one or more pairs of sister chromatids fail to disjoin during mitosis and a strand of chromosome is ‘trapped’ in the region of cytokinesis. The fate of most such bridges, if not resolved, is the degradation of the DNA within and thus the development of chromosomal aneuploidy [44]. Anaphase bridges have been shown to arise from several defined mechanisms, most often involving a lack of proper telomere maintenance [38] sometimes involving topoisomerase function [45] or, helicase [46] function. Two publications from the same group offer evidence that RAP treatment can lead to chromosomal non-disjunction during M-phase in yeast and mammalian cell cultures [47], [48]. Strikingly, estradiol treatment consistently *decreased* anaphase bridges in mitotic SIGC versus cells treated with the corresponding concentration of RAP alone. Since estradiol can counter the effects of RAP, we propose that both the cell cycle effects and the induction of mitotic anomalies act to control the rate of follicle growth directly and may be related to the selection and maintenance of the dominant follicle (below).

It has been reported that growing follicles in a variety of mammalian species contain high proportions of aneuploid granulosa cells [39], [40]. The largest (dominant) follicles are a notable exception, as those appear to be nearly uniformly euploid [41]. Since the dominant follicle itself produces high levels of estradiol (likely the *highest* levels relative to subordinate follicles) [49], [49]–[54], it is possible that mTOR is involved in the process of dominant follicle selection in this fashion. These data in sum further support the TOR pathway as a crucial factor in the control of the production of fertilizable eggs.

## Materials and Methods

### Mice/Ethics Statement

The studies included in this work were performed in accordance with the Yale University Institutional Animal Care and Use Committee Policies for Animal Use under an approved animal protocol (#2005-10990). Handling and euthanasia of mice were performed per the National Institutes of Health Guide for the Care and Use of Laboratory Animals. 8-week old C57BL/6 mice were used for these studies.

### Cells/cell culture

SIGC cells were maintained in DMEM/F12 with 10% serum, with 1× Penicillin/Streptomycin. 50000 cells per well were plated in 96-well plates. Cell viability was assessed *via* the CellTiter96 assay (Promega) at 7 hour intervals after the addition of RAP (LC Laboratories), estradiol, or corresponding vehicle volumes of ethanol. 10 ng/ml estradiol was used to mimic intrafollicular concentration after [55]. Where indicated, the estradiol receptor antagonist compound ICI 182780 (Tocris Bioscience). For direct counts of mitotic figures, cells were plated in 8-well culture chambers and treated identically with RAP or vehicle. 21 hours after treatment, cells were labeled with either DAPI or TO-PRO-3 and visualized after image capture at 100× magnification.

### Immunostaining

Cells grown in slide chambers were fixed, washed, and blocked in antibody buffer: Tris-Buffered Saline containing 0.1% Tween-20, 5% normal goat serum, and 0.01% Tween-20 then incubated with rabbit anti-mTOR, anti-P-mTOR, anti-Raptor, anti-phospho Thr 37/46 4E-BP (each from Cell Signaling Technology, Inc. #'s #2983, #2971, #4978, and #2855, respectively, and diluted 1∶300), or anti phospho Thr389 p70S66 kinase (Sigma, #S6311) overnight at 4

C. Cells were then washed 3 times for 10 minutes, and stained with anti-rabbit second antibody (Alexa 546-conjugate, Molecular Probes). Cells were optionally co-stained with Alexa 488-conjugated anti 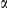-tubulin or anti-phospho Histone H3 (Molecular Probes). Cells were then washed with PBS 3 times for 5 minutes, and cell nuclei were stained by including DAPI in the second PBS wash. Cells were coverslipped in Crystal Mount mounting media. Images were captured using a Zeiss Axioplan 2 epifluorescence microscope, an in-line digital camera, and Axiovision imaging software.

### Western blot analysis

For analysis of intracellular proteins, cells were lysed using 1% NP40 and 0.1% SDS in the presence of protease and phosphatase inhibitors. Protein concentrations were calculated by BCA assay (Pierce Biotechnology, Rockford, IL). Proteins were then diluted with gel loading buffer to 20 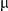
g and boiled for 5 min. Proteins were resolved under reducing conditions on either 10 or 12% SDS-PAGE gels and then transferred onto nitrocellulose membranes (NEN Life Sciences, Boston, MA). Membranes were blocked in 5% powdered milk in PBS/0.05% Tween 20 (PBS-T), then washed and incubated overnight at 4

C with anti-Phospho-Ser 2448 mTOR or anti-Phospho-Thr389 p70S6K in PBS-T/1% milk. After washing, second antibody conjugated to peroxidase (Vector Laboratories) in PBS-T/1% milk was applied and signal detected by enhanced chemiluminescence (NEN Life Sciences). All experiments were repeated at least three times, and the intensity of the signals was analyzed using a digital imaging analysis system and 1D Image Analysis Software (Scientific Imaging Kodak Company). 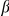-actin (mouse ant-i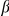-actin, Sigma) was used as internal control.

### Cell counts and cell viability/apoptosis analysis

5000 cells were grown in wells of a 12-well plate as above with either ethanol vehicle, or the specified concentration of RAP and/or estradiol in triplicate. At the end of culture periods, cells were trypsinized, and resuspended in 100 ul of PBS containing 0.01% Trypan Blue (EMD Chemicals); the number of cells was determined using a hemocytometer by counting those cells that excluded Trypan Blue. Cell viability (and indirectly, cell death,) was assessed using the CellTiter assay [56], [57] 18, 24, or 48 hours after the addition of vehicle or RAP. Caspase activity was measured using Caspase-GLO assays per [58].

### Flow cytometric cell cycle analysis

For flow cytometric analysis, cells were grown in the indicated conditions, fixed in 70% Ethanol, and stained with Propidium iodide per standard protocols. Sample runs and analyses were performed using a FACScan cytometer in the Yale FACS facility, Section of Immunobiology, Department of Internal Medicine and Yale Cancer Center.

### Ovulation assessment, histomorphometric analysis of mitotic figures, and in vitro fertilization

For *in vivo* studies, 8 week old C57Bl/6 animals were injected intraperitoneally with the indicated doses of RAP or vehicle as follows during a ‘superovulation regime’ and ovaries were isolated. Injections of RAP (5 or 50 mg/kg body weight) or vehicle were given over two days. On Day 1, 5 IU PMSG was administered in the afternoon. Two drug or vehicle doses followed on Day 2 and Day 3; 5 IU hCG was injected into animals the afternoon of Day 3. The morning of predicted ovulation (14 hours post-hCG), animals were killed and oocytes were flushed from oviducts and counted. Where indicated, *in vitro* fertilization studies were performed per Hogan et al, 1994. Briefly, the morning of egg collection, a male C57Bl/6 animal was killed and capacitated sperm was added to collected eggs. The determination of anaphase/telophase mitotic configuration in growing mouse ovarian follicles was performed as follows: three large early antral follicles with no visible apoptotic granulosa cells were selected at random in three unique ovaries; mitotic figures were counted from end-to-end in serial sections of each follicle. Ovaries were fixed in Dietrich's fixative, stained with Weigert's Iron Hematoxylin and Picric acid/Methyl Blue and mounted on glass slides. All samples were counted blindly, on coded slides [42].

### Statistical analyses

All statistical analyses were performed using the GraphPad Prism 4 statistical package (GraphPad Software, Inc.). Mean data is presented for each set of replicates (number of replicates per trial is indicated for each experiment), and error bars represent the SEM. One-way ANOVA analysis was performed upon densitometric data from Western blots, and two-way ANOVA was performed upon data from biochemical viability, cell number, and anaphase bridge quantitation experiments. P values are listed per experiment to denote statistical significance.

## Supporting Information

Figure S1 **P-Ser 2448 mTOR and Raptor exhibit overlapping expression in SIGC during mitosis.** Immunofluorescence detection of P-mTOR (A) and Raptor (B) in SIGC show that the phosphoprotein and mTORC1 cofactor are each present at high levels during mitosis. While P-mTOR is present at high levels between late G2/early prometaphase (A, green arrow) and late telophase/nearly complete cytokinesis (A, yellow arrowhead), Raptor protein is at high levels between late G2/prometaphase (B, green arrow) and approximately anaphase. Some anaphase cells show high Raptor expression (dual yellow arrowheads, compare to P-mTOR in A) while other cells show lower Raptor expression (white arrowheads).
(TIF)

Figure S2 **Scheme of superovulation.** 8-week old mice received gonadotrophin- containing preparations (PMSG and hCG) and either RAP or VEH, after which eggs were collected, evaluated, and subjected to *in vitro* fertilization.
(TIF)
