# Supplementary figures and images for: mTOR Controls Ovarian Follicle Growth by Regulating Granulosa Cell Proliferation
Source: PLoS One. 2011 Jul 5;6(7):e21415. doi: 10.1371/journal.pone.0021415 (PMC3130037; doi:10.1371/journal.pone.0021415)

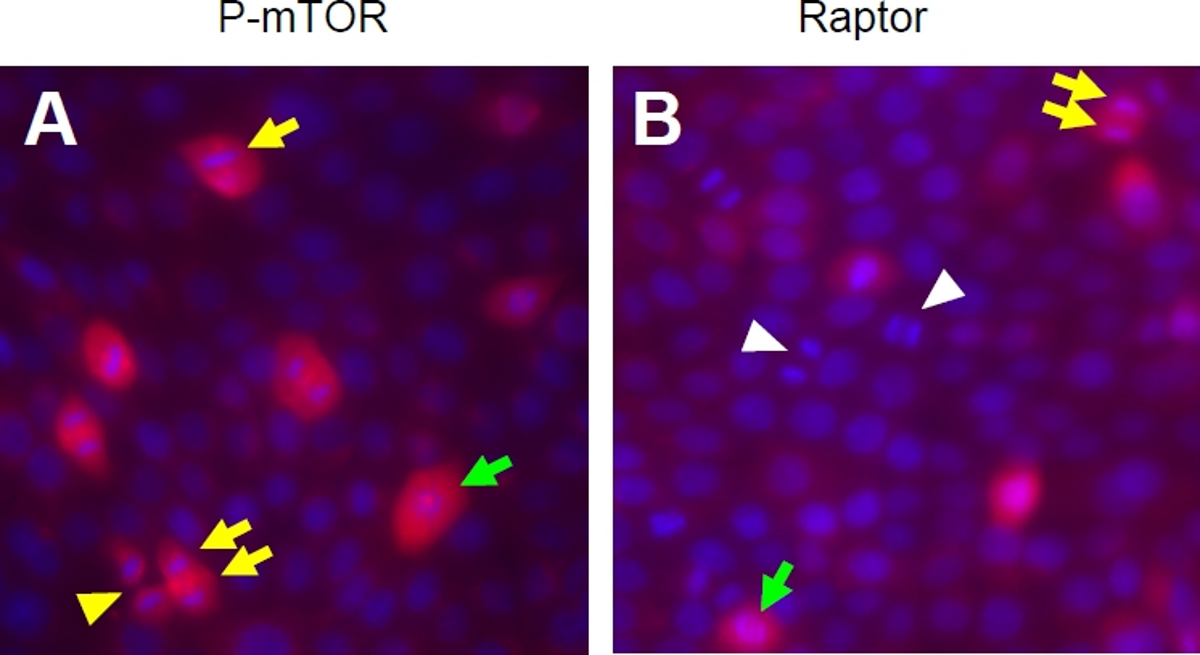

Supplement: Figure S1 — P-Ser 2448 mTOR and Raptor exhibit overlapping expression in SIGC during mitosis. Immunofluorescence detection of P-mTOR (A) and Raptor (B) in SIGC show that the phosphoprotein and mTORC1 cofactor are each present at high levels during mitosis. While P-mTOR is present at high levels between late G2/early prometaphase (A, green arrow) and late telophase/nearly complete cytokinesis (A, yellow arrowhead), Raptor protein is at high levels between late G2/prometaphase (B, green arrow) and approximately anaphase. Some anaphase cells show high Raptor expression (dual yellow arrowheads, compare to P-mTOR in A) while other cells show lower Raptor expression (white arrowheads). (TIF) [file pone.0021415.s001.tif]

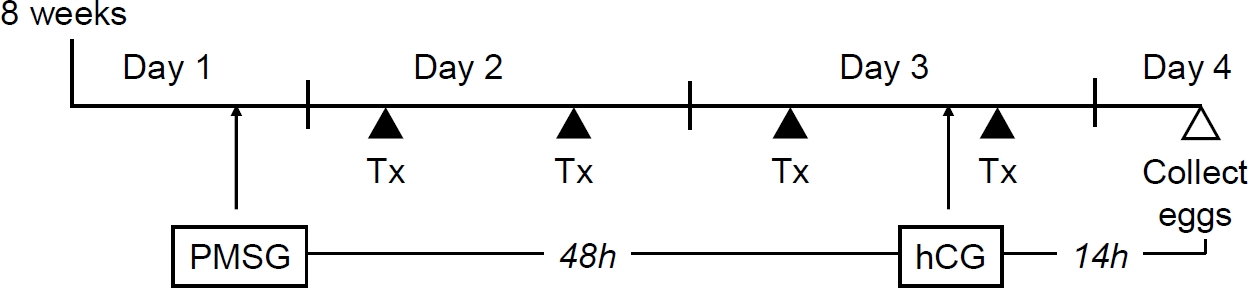

Supplement: Figure S2 — Scheme of superovulation. 8-week old mice received gonadotrophin- containing preparations (PMSG and hCG) and either RAP or VEH, after which eggs were collected, evaluated, and subjected to in vitro fertilization. (TIF) [file pone.0021415.s002.tif]
